# A lower psoas muscle index predicts a poorer prognosis in metastatic hormone‐naïve prostate cancer

**DOI:** 10.1002/bco2.36

**Published:** 2020-08-13

**Authors:** Genta Iwamoto, Takashi Kawahara, Toshitaka Miyai, Masato Yasui, Hisashi Hasumi, Yasuhide Miyoshi, Masahiro Yao, Hiroji Uemura

**Affiliations:** ^1^ Department of Urology and Renal Transportation Yokohama City University Medical Center Yokohama Japan; ^2^ Department of Urology Yokohama City University Graduate School of Medicine Yokohama Japan

**Keywords:** prostate, psoas muscle index, sarcopenia

## Abstract

**Introduction:**

A recent investigation revealed that sarcopenia was associated with a poorer prognosis in some solid malignancies, including prostate cancer. In most reports, sarcopenia was defined as a low psoas volume on CT. This study investigated the association of sarcopenia, determined according to the psoas muscle volume and density on CT, with the prognosis in patients with metastatic hormone‐naïve prostate cancer (mHNPC).

**Methods:**

A total of 66 patients initially diagnosed with mHNPC were enrolled in this study. Skeletal muscle was evaluated according to the psoas muscle index density (PMID) on computed tomography scans. The psoas muscle volume was calculated at the level of L3 and CT density was evaluated as the mean CT density at the psoas muscle area. We divided the patients into higher and lower PMID groups.

**Results:**

The lower PMID group (on both sides) showed a poorer overall survival than the higher PMID group (Right: 32.5 vs 99.0 months in Rt PMID, *P* = .014; Left: 36.0 vs 100.0 months in Lt PMID, *P* = .029). The lower PMID group (on both sides) showed a shorter time to CRPC (Right: 9.0 vs 42.0 months in Rt PMID, *P* = .006; Left: 9.0 vs 31.0 months in Lt PMID, *P* = .005). A multivariate analysis showed that lower Rt PMID and Lt PMID were independent risk factors for poorer OS (HR:2.02, 95%CI: 1.04‐3.90, *P* = .037, HR:2.29, 95%CI: 1.18‐4.47, *P* = .015, respectively). For CRPC, both Rt and Lt lower PMID also showed independent risk factors for shorter time to CRPC (HR:2.39, 95%CI: 1.23‐4.62, *P* = .010, HR:2.43, 95%CI: 1.23‐4.78, *P* = .010, respectively).

**Conclusions:**

Among mHNPC patients, both lower PMID groups showed a poorer overall survival and shorter time to CRPC than the higher PMID groups.

## 1 INTRODUCTION

In 1989, Rosenberg suggested the concept of sarcopenia as decrease of skeletal muscle.^1^ The term sarcopenia was coined as a combination of the words “sarx” and “penia.” Recently, “sarcopenia” has been used to describe a decrease in skeletal muscle due to various reasons, in addition to the decrease associated with aging in elderly individuals. In 2010, the European Working Group on Sarcopenia in Older people (EWGSOP) recommended using both a low muscle mass and low muscle function (strength or performance) to define sarcopenia.^2^ The Asian working group for sarcopenia (AWGS) also suggested a definition of sarcopenia in 2014.^3^ Because the skeletal volume is not strictly correlated with muscle strength, the EWFSOP and AWGS included muscle strength in the definition of sarcopenia.^3^

The presence of malignant disease was thought to be a reason for the development of sarcopenia and a recent study suggested that systemic chemotherapy and radiotherapy were associated with decreased muscle volume and influenced the prognosis of malignant diseases.^4^, ^5^ Previous sarcopenia‐related studies have evaluated the area or volume of the psoas muscle and investigated the influence on the prognosis.^6^ For pancreatic malignancy, the psoas area and density‐predicted prognosis were shown to be independent risk factors for the prognosis.^7^ However, the qualitative of psoas muscle has not been examined in prostate cancer.^7^, ^8^

In the present study, we examined the importance of the psoas muscle index density (PMID) as a qualitative prognostic factor in metastatic hormone‐naïve prostate cancer (mHNPC).

## 2 MATERIALS AND METHODS

A total of 66 patients with mHNPC and who were managed in Yokohama City University Medical Center (Yokohama, JAPAN) between 2008 and 2014 were enrolled in this study. All patients had de novo metastatic prostate cancer and received androgen deprivation therapy without up‐front abiraterone or docetaxel. The exclusion criterion was enrollment in clinical trial for initial mHNPC treatment. CT scans were used for calculating the prostate muscle area. The psoas muscle index (PMI) was calculated as the right/left area at the level of L3 divided by the square of body height. The PMID was calculated multiplied between PMI and CT density in the psoas muscle area (Figure S1).

Receiver operator characteristic curves (ROCs) were used to determine the candidate cutoff points. For ROCs, we set the event as death at 4 years after the diagnosis and castration‐resistant prostate cancer (CRPC) at 3 years after the diagnosis. The time to CRPC and the OS were analyzed using the Kaplan‐Meier method, and the resultant curves were statistically tested by the log‐rank method. A Cox proportional hazards model was used for univariable and multivariable analysis. Multivariable analysis was performed using the factors which showed significant differences by univariable analysis. We also performed multivariable analysis using important clinical factors including Gleason Score, lactate dehydrogenase (LDH), and Rt PMID. *P* values of <.05 were considered to indicate statistical significance in all statistical analyses.

## 3 RESULTS

A total of 66 patients were enrolled in this study. This study was carried out in compliance with the Declaration of Helsinki and was approved by the Institutional Review Board of Yokohama City University Hospital (D1507018).

The patients’ characteristics, including the age, Gleason score, presence of visceral metastasis, presence of lymph node metastasis, presence of symptoms, performance status, albumin (Alb), alkaline phosphatase (ALP), hemoglobin (Hb), creatinine (Cre), prostate‐specific antigen (PSA), LDH, and PMID, are summarized in Table 1. The Gleason score was pathologically confirmed by a prostate needle biopsy.

**TABLE 1:** Patients' background

Variables	Median (mean ± SD), n (%)			*P* value
	All	Low Rt PMID	High Rt PMID	
Number of patients	66	36	30	
Age (y)	72 (71.6 ± 7.7)	72 (72.2 ± 7.3)	69 (70.9 ± 8.2)	.511
Symptomatic	44 (66.7%)	22 (61.1%)	22 (73.3%)	.294
Gleason score				
<8	8 (12.1%)	4 (11.1%)	4 (13.3%)	.655
8	32 (48.5%)	16 (44.4%)	16 (53.3%)	
>8	26 (39.4%)	16 (44.4%)	10 (33.3%)	
Visceral metastasis	13 (19.7%)	5 (13.9%)	8 (26.7%)	.194
Lymph node metastasis	39 (59.1%)	21 (58.3%)	18 (60.0%)	.891
Performance status				
0	15 (22.7%)	8 (22.2%)	7 (23.3%)	.368
1	30 (45.5%)	14 (38.9%)	16 (53.3%)	
2	10 (15.2%)	5 (13.9%)	5 (16.7%)	
3	10 (!5.2%)	8 (22.2%)	2 (6.7%)	
Unknown	1 (1.5%)	1 (2.8%)	0 (0.0%)	
Albumin	4.2 (4.1 ± 0.5)	4.1 (4.0 ± 0.5)	4.3 (4.3 ± 0.4)	.019
Alkaline phosphatase	526.0 (816.3 ± 1003.6)	571 (1024.5 ± 1267.9)	487 (565.1 ± 441.1)	.052
Hemoglobin	13.1 (12.7 ± 2.1)	13.0 (12.4 ± 2.1)	13.1 (12.9 ± 2.0)	.331
Creatinine	0.9 (1.1 ± 0.8)	0.8 (1.0 ± 0.7)	0.9 (1.2 ± 1.0)	.558
PSA	261.0 (807.6 ± 1009.5)	326.2 (846.5 ± 966.2)	203.0 (762.2 ± 1072.6)	.742
Lactate dehydrogenase	206.5 (239.9 ± 112.3)	214 (261.3 ± 139.2)	201 (214.2 ± 60.0)	.077
Body mass index	22.9 (22.4 ± 5.6)	21.7 (21.6 ± 5.5)	23.6 (23.4 ± 5.8)	.212
Docetaxel treatment	23 (34.8%)	8 (22.2%)	15 (50.0%)	.018
ENZ/ABI treatment	29 (43.9%)	19 (52.8%)	10 (33.3%)	.113
Complication				
Hypertension	20 (30.3%)	14 (38.9%)	6 (20.0%)	.096
Diabetes mellitus	11 (16.7%)	5 (13.9%)	6 (20.0%)	.507
Hyper lipidemia	8 (12.1%)	4 (11.1%)	4 (13.3%)	.783
Other cancer	10 (15.2%)	5 (13.9%)	3 (10.0%)	.63

Abbreviations: ABI, abiraterone acetate; ENZ, enzalutamide; PMID, psoas muscle index density; PSA, prostate specific antigen.

**FIGURE 1:**
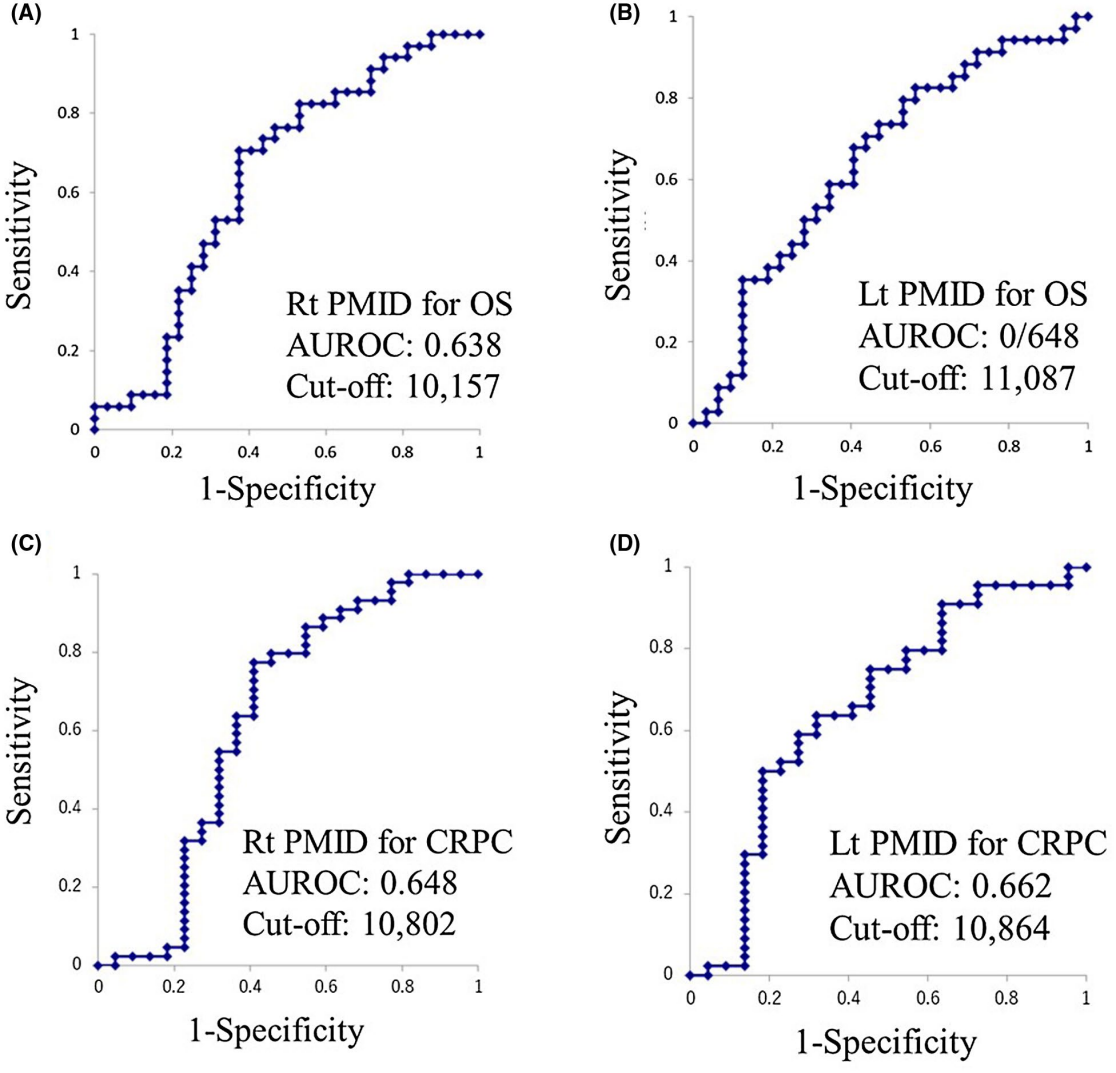
A receiver operator characteristic curve analysis revealed the optimal cutoff values. (A) Rt PMID for the overall survival, (B) Lt PMID for the overall survival, (C) Rt PMID for CRPC, (D) Lt PMID for CRPC

**FIGURE 2:**
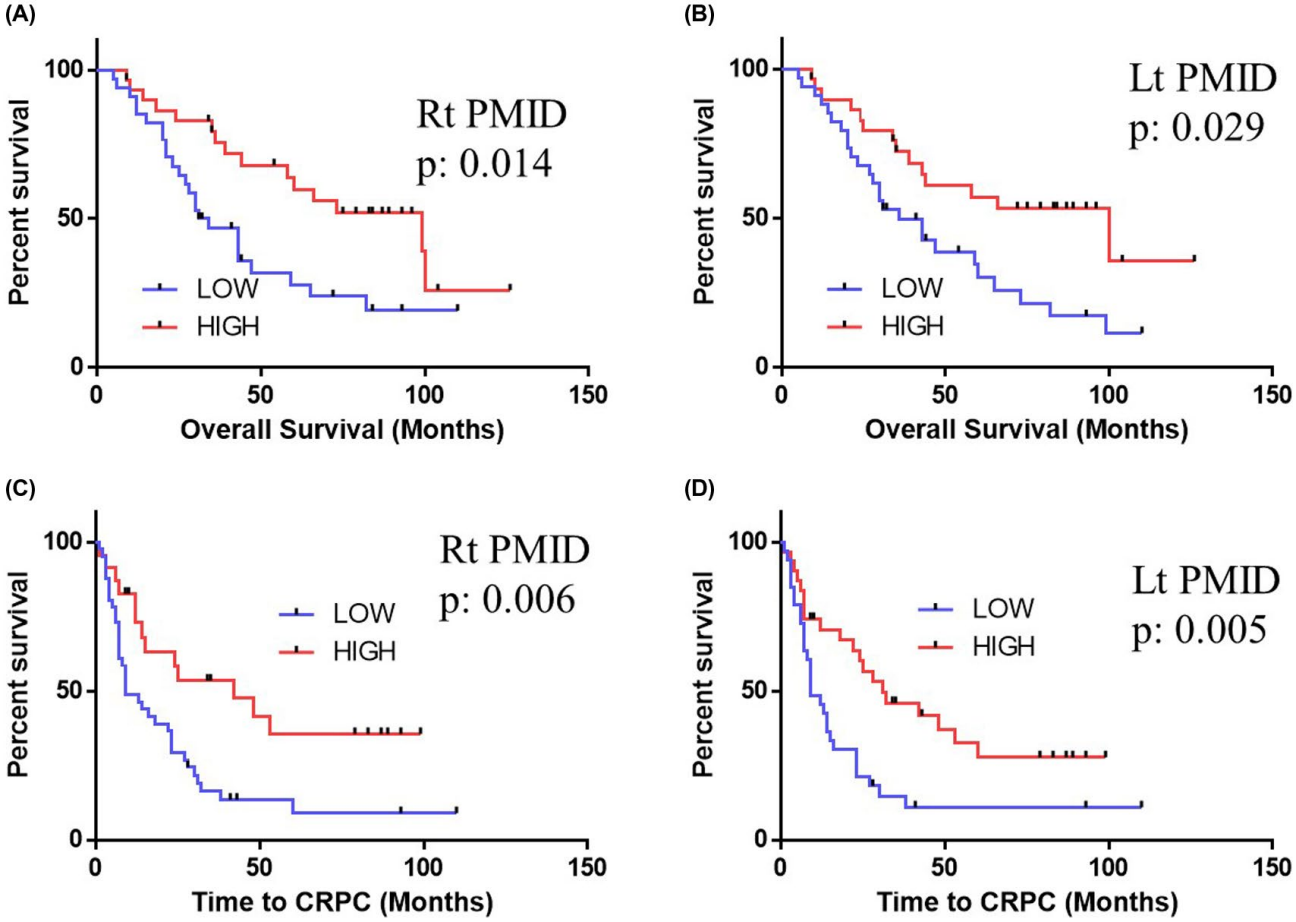
The Kaplan‐Meier curves for the overall survival and CRPC. (A) Rt PMID for the overall survival, (B) Lt PMID for the overall survival, (C) Rt PMID for CRPC, (D) Lt PMID for CRPC

The median (mean ± SD) age of the patients was 72 years old (71.6 ± 7.7 years old), and 26 (39.4%) patients had an initial Gleason Score of 9 or 10. Lymph node metastasis was found in 39 (59.1%) and visceral metastasis in 13 (19.7%). Among these 66 patients, 49 (74.2%) developed CRPC, and 40 (60.6%) died. Regarding the CRPC status, 21 of 49 (42.9%) received second‐generation antiandrogen drugs (enzalutamide and/or abiraterone), and 27 of 49 (55.1%) received docetaxel. The ROCs drawn using the left PMID had an area under the ROC curve (AUC) of 0.734, and the candidate cutoff value was 11 862.2. ROCs showed respective AUCs and candidate cutoff points of 0.638 and 10 157 (Rt PMID for OS), 0.648 and 11 087 (Lt PMID for OS), 0.648 and 10 802 (Rt PMID for CRPC), and 0.662 and 10 864 (Lt PMID for CRPC) (Figure 1). There were no marked differences between the higher and lower CT density groups (Figure S2). As shown in Figure 2, the respective median OS values in the lower vs higher PMID groups were 32.5 vs 99 months in Rt PMID (*P* = .014) and 36 vs 100 months in Lt PMID (*P* = .029). The respective median durations to CRPC in the lower vs higher PMID groups were 9 vs 42 months in Rt PMID (*P* = .006) and 9 vs 31 months in Lt PMID (*P* = .005) (*P* = .016).

A multivariate analysis showed that both lower Rt and Lt PMID were an independent risk factor for a poorer OS (hazard ratio [HR]: 2.02, 95% confidence interval [CI]: 1.04‐3.90, *P* = .037, HR:2.29 95%CI: 1.18‐4.45, *P* = .015, respectively) (Table 2). For CRPC, both lower Rt and lower Lt PMID were also independent risk factors associated with a shorter time to CRPC (HR: 2.39, 95% CI: 1.23‐4.62, *P* = .010, HR: 2.43, 95% CI: 1.23‐4.78, *P* = .010, respectively) (Table 3). We also performed multivariable analysis using clinical important risk factors including Gleason Score, LDH, and Rt PMID and lower Rt PMID showed significant poorer prognostic factor (HR: 2.06, 95% CI: 1.05‐4.04, *P* = .035) (Table S1).

**TABLE 2:** Univariable and multivariable analyses for overall survival

Variables	Univariable				Multivariable				Multivariable			
	HR	95%CI		*P* value	HR	95%CI		*P* value	HR	95%CI		*P* value
		Lower	Upper			Lower	Upper			Lower	Upper	
Age ≥72 vs <72	0.81	0.44	1.52	.517								
Gleason score ≥8 vs <8	1.57	0.56	4.45	.391								
Visceral metastasis Yes vs No	1.12	0.52	2.45	.770								
Lymph node metastasis Yes vs No	1.22	0.64	2.32	.540								
Symptomatic vs Non‐symptomatic	1.78	0.74	4.29	.200								
Performance status ≥2 vs <2	1.98	1.02	3.83	.043	1.82	0.93	3.54	.078	2.10	1.08	4.08	.029
Alb ≥4.2 vs <4.3	1.08	0.56	2.09	.819								
ALP ≥495 vs <495	1.49	0.78	2.84	.223								
Hb <13.1, ≥13.1	1.45	0.75	2.80	.273								
Cre ≥0.87 vs <0.87	1.53	0.78	2.99	.215								
PSA ≥288.6 vs <288.6	0.88	0.47	1.65	.700								
LDH ≥202 vs <202	1.64	0.87	3.12	.128								
DOC yes vs no	0.86	0.45	1.63	.638								
ENZ/ABI yes vs no	1.72	0.92	3.23	.090								
RtPMID <10 157 vs ≥10 157	2.20	1.15	4.22	.017	2.02	1.04	3.90	.037				
LtPMID <11 087 vs ≥11 087	2.25	1.17	4.33	.015					2.29	1.18	4.45	.015

Abbreviations: Alb, albumin; ALP, Alkaline phosphatase; Cre, creatinine; Hb, hemoglobin; LDH, lactate dehydrogenase; PMID, psoas muscle index density; PSA, prostate‐specific antigen.

**TABLE 3:** Univariable and multivariable analyses for time to CRPC

Variables	Univariable				Multivariable				Multivariable			
	HR	95%CI		*P* value	HR	95%CI		*P* value	HR	95%CI		*P* value
		Lower	Upper			Lower	Upper			Lower	Upper	
Age ≥72 vs <72	0.55	0.31	0.98	.043	0.63	0.31	1.27	.198	0.67	0.34	1.34	.263
Gleason score ≥8 vs < 8	3.57	1.10	11.63	.034	2.15	0.63	7.34	.222	2.02	0.58	7.07	.269
Visceral metastasis Yes vs No	0.87	0.42	1.79	.702								
Lymph node metastasis Yes vs No	1.17	0.65	2.08	.602								
Symptomatic vs Non‐symptomatic	2.33	1.03	5.23	.041	2.25	0.94	5.42	.070	2.46	1.01	6.00	.049
Performance status ≥2 vs <2	1.22	0.67	2.30.	.524								
Alb ≥4.2 vs <4.3	0.98	0.54	1.75	.933								
ALP ≥495 vs <495	1.54	0.86	2.73	.145								
Hb <13.1, ≥13.1	1.48	0.82	2.69	.193								
Cre ≥0.87 vs <0.87	1.11	0.62	2.00	.718								
PSA ≥288.6 vs <288.6	1.19	0.68	2.09	.543								
LDH ≥202 vs <202	1.19	0.67	2.12	.555								
RtPMID <10 862 vs ≥10 862	2.05	1.08	3.89	.028	2.39	1.23	4.62	.010				
LtPMID <10 864 vs ≥10 864	2.26	1.26	4.06	.007					2.43	1.23	4.78	.010

Abbreviations: Alb, albumin; ALP, Alkaline phosphatase; Cre, creatinine; Hb, hemoglobin; LDH, lactate dehydrogenase; PMID, psoas muscle index density; PSA, prostate‐specific antigen.

## 4 DISCUSSION

Malignant disease is sometimes thought to be a secondary cause of sarcopenia. Patients with malignant disease show elevated IL‐6 or TNF‐alpha levels, which introduces protein catabolism and decomposition. A worse nutrition condition or lower activity suppresses protein synthesis, and results in a tendency to develop sarcopenia.^4^, ^5^

This study first examined the importance of the muscle density as a prognostic factor in mHNPC patients. The circumference of the arm, dual‐energy X‐ray absorptiometry (DEXA), and bioelectrical impedance analysis (BIA) and imaging (eg, CT or MRI) findings are used as a reflection of muscle power. Previous reports have usually used CT, as CT scans are easy to analyze retrospectively. In particular, at the L3 level, the psoas muscle volume has been reported to be correlated with the whole‐muscle volume, and the PMI is determined as the psoas muscle area divided by the square of body height.^9^ However, the PMI does not reflect the muscle power. In pancreatic malignancy, the psoas area and density predicted prognosis were shown to be independent risk factors for the prognosis.^7^ Thus, the present study used PMID to reflect the muscle power. We used the PMID as a multiplier between PMI and the CT density and a lower PMID was related to a poorer prognosis in patients with mHNPC. Chakedis et al and Namn et al revealed the importance of the psoas muscle density on CT in patients with biliary tract cancers and pancreatic cancer.^7^, ^8^

Several limitations associated with the present study warrant mention. First, it was a nonrandomized retrospective study and enrolled a relatively small number of patients, so a further prospective study is needed in order to confirm these results. Second, we were unable to clarify the mechanism underlying the relationship between low PMID and a poor prognosis. A previous report suggested the involvement of systemic inflammation and sarcopenia.^4^, ^5^ While this study was unable to confirm the relevant mechanism, sarcopenia might influence advanced prostate cancer development. Third, the HR in each side of PMID showed differences. Our previous report also revealed that the both psoas muscle volume were correlated.^10^ Though the meanings of differences between right and left, both PMID would be a useful factors to predict prognosis. Further study is needed to confirm the HR by lower PMID.

Recent studies have identified sarcopenia (loss of skeletal muscle mass) as one of the important factors for poorer prognostic factors.^11^ The PMI, which was a relatively simple method to represent skeletal muscle volume in whole body. This index is attracting attentions as one of the prognostic factors for malignancy, although it was popularized as an index to quantify sarcopenia at first. Based on the findings of the present study, sarcopenia would be associated with a poor prognosis in mHNPC patients. Psoas muscle volume and muscle CT density might be clues that can be used to predict the prognosis of patients with mHNPC.

## CONFLICT OF INTEREST

The authors declare no conflicts of interest in association with the present study.

## ETHICS

This study was carried out in compliance with the Declaration of Helsinki and was approved by the Institutional Review Board of Yokohama City University Hospital (D1507018).

## Supporting information

Fig S1

Fig S2

Table S1

## Data Availability

Due to ethical restrictions, the raw data underlying this paper are available upon request from the corresponding author.
