# Supplementary figures and images for: A lower psoas muscle index predicts a poorer prognosis in metastatic hormone‐naïve prostate cancer
Source: BJUI Compass. 2020 Aug 13;2(1):39–45. doi: 10.1002/bco2.36 (PMC8988845; doi:10.1002/bco2.36)

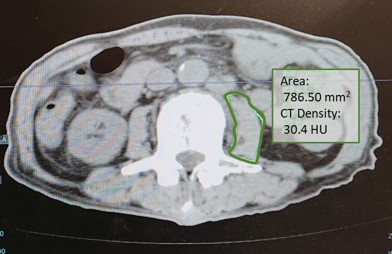

Supplement: Supplementary file 1 — Fig S1 [file BCO2-2-39-s002.jpg]

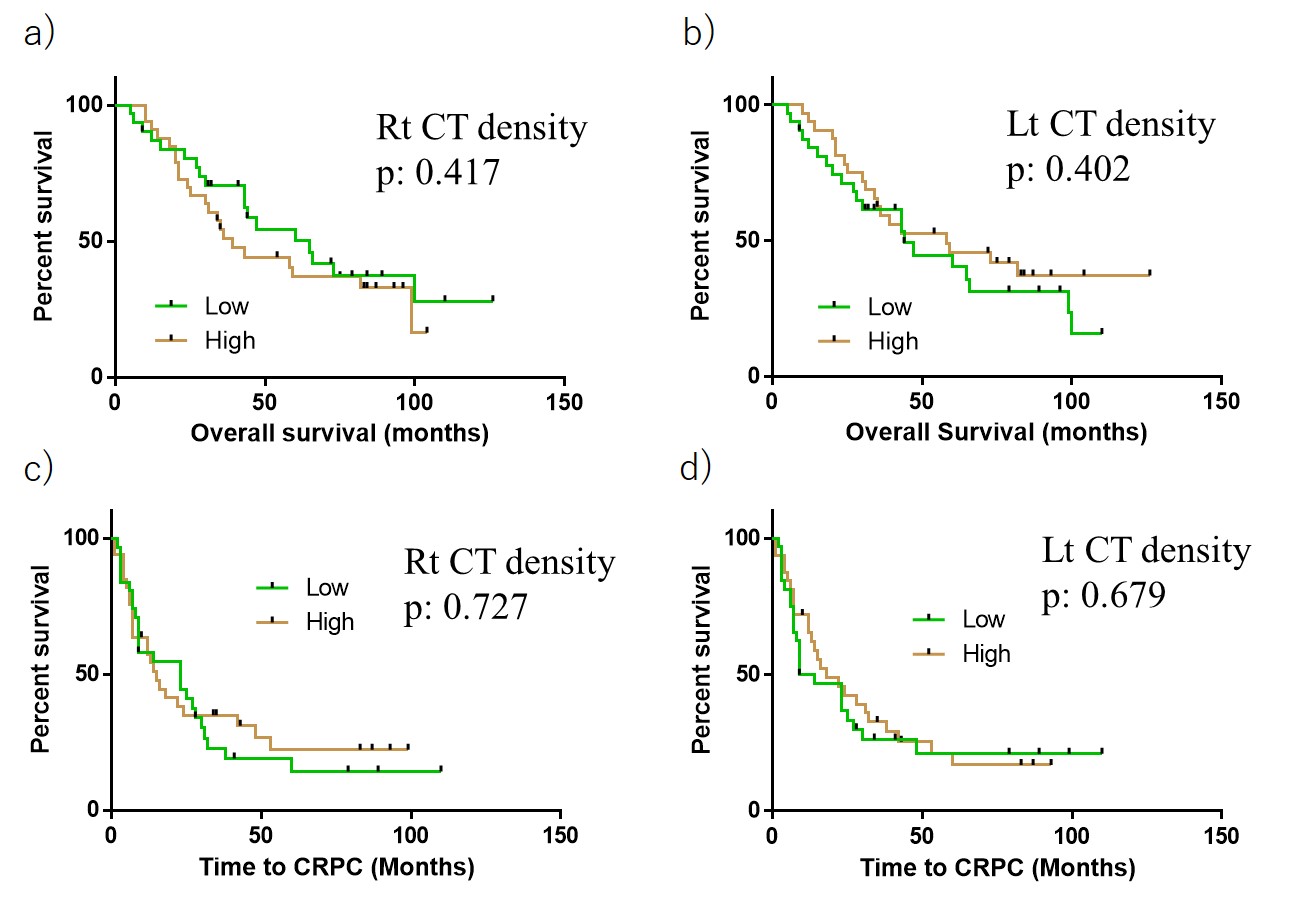

Supplement: Supplementary file 2 — Fig S2 [file BCO2-2-39-s001.jpg]
