# Supplementary material for: A lower psoas muscle index predicts a poorer prognosis in metastatic hormone‐naïve prostate cancer
Source: BJUI Compass. 2020 Aug 13;2(1):39–45. doi: 10.1002/bco2.36 (PMC8988845; doi:10.1002/bco2.36)
Supplement: Supplementary file 3 — Table S1 [file BCO2-2-39-s003.docx]

| Supplementary Table: Multivariable analyses for overall survival | | | | |
| --- | --- | --- | --- | --- |
| Variables | Multivariable | | | |
|  | HR | 95%CI | | p value |
|  |  | Lower | Upper |  |
| Gleason Score ≥8 vs<8 | 1.50 | 0.53 | 4.22 | 0.451 |
| LDH ≥202 vs <202 | 1.47 | 0.77 | 2.81 | 0.240 |
| Rt PMID <10,157 vs ≥10,157 | 2.06 | 1.05 | 4.04 | 0.035 |
| LDH: Lactate dehydrogenase, PMID: psoas muscle index density | | | | |
